# Supplementary material for: Multi-area recordings and optogenetics in the awake, behaving marmoset
Source: Nat Commun. 2023 Feb 2;14:577. doi: 10.1038/s41467-023-36217-5 (PMC9895452; doi:10.1038/s41467-023-36217-5)
Supplement: Supplementary file 1 — Supplementary Information [file 41467_2023_36217_MOESM1_ESM.pdf]

# Supplementary Material

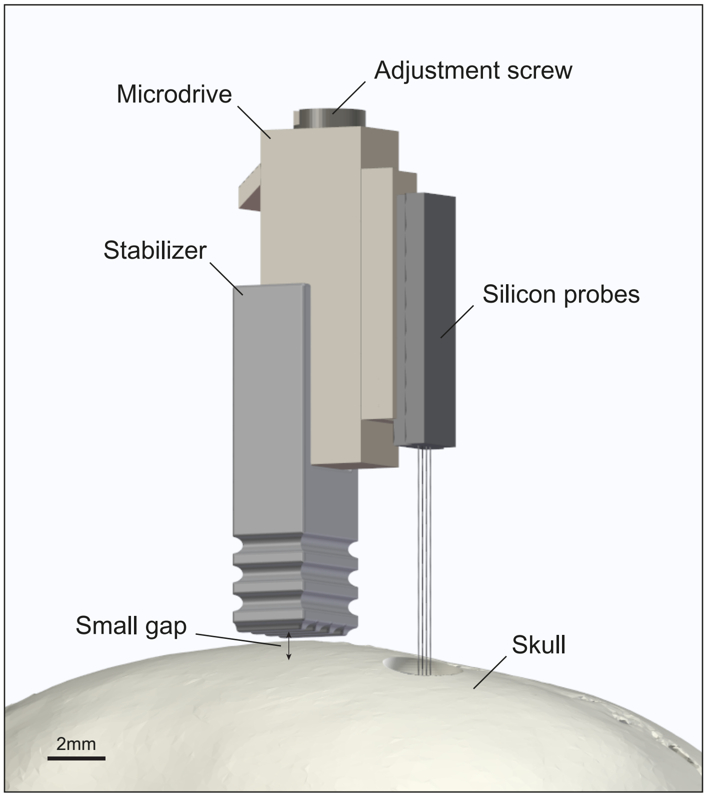


**Supplementary Figure 1 | Close-up rendering of the stabilizer and microdrive assembly.** The length of the silicon probes (≈8mm) results in a gap between the bottom of the microdrive and the skull when the probes are implanted into the superficial parts of the brain. Therefore, a 3D-printed titanium stabilizer was glued to the microdrive prior to the surgery. During the implantation, the assembly can be positioned very close to the skull, such that the gap that needs to be filled with cement is very small.

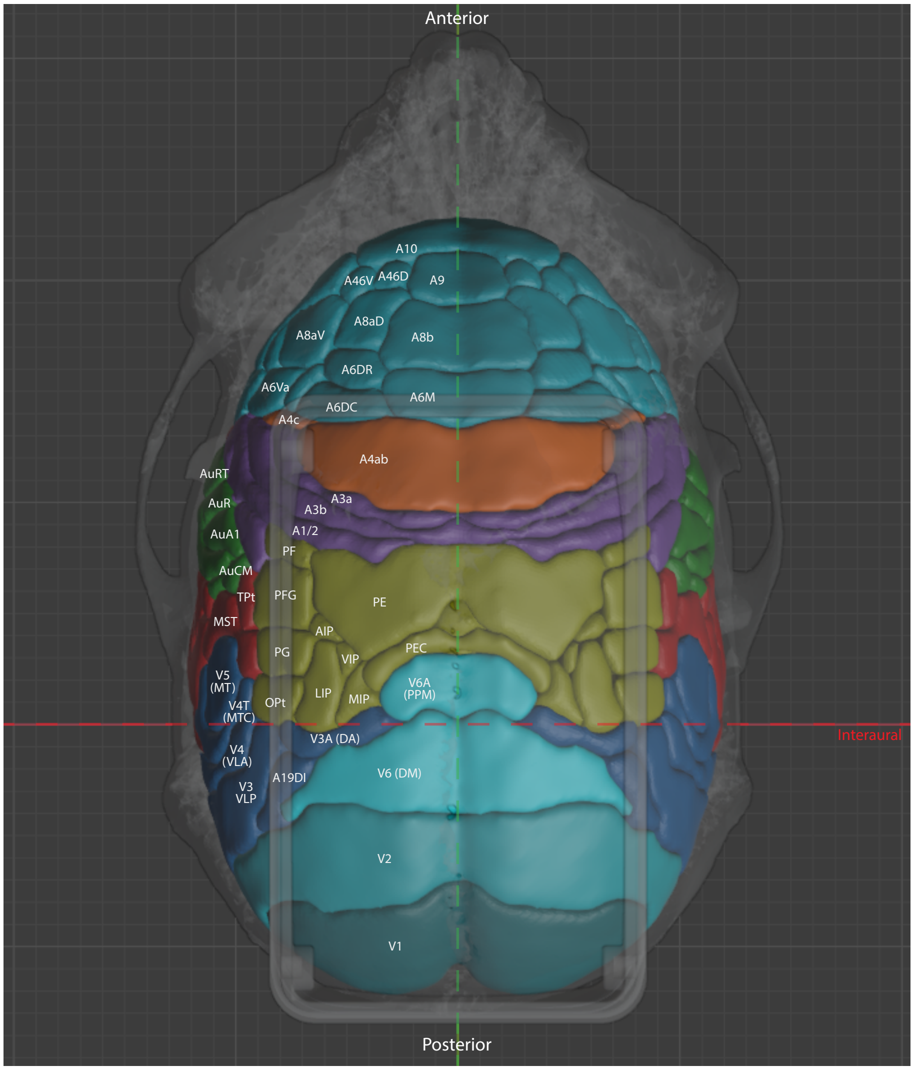


**Supplementary Figure 2 | Accessible cortical areas.** Top view of the chamber and cortical brain areas directly underneath. Red dashed line indicates interaural axis. Green dashed line indicates anterior-posterior axis. Area segmentation and labels from Liu et al. (2018).

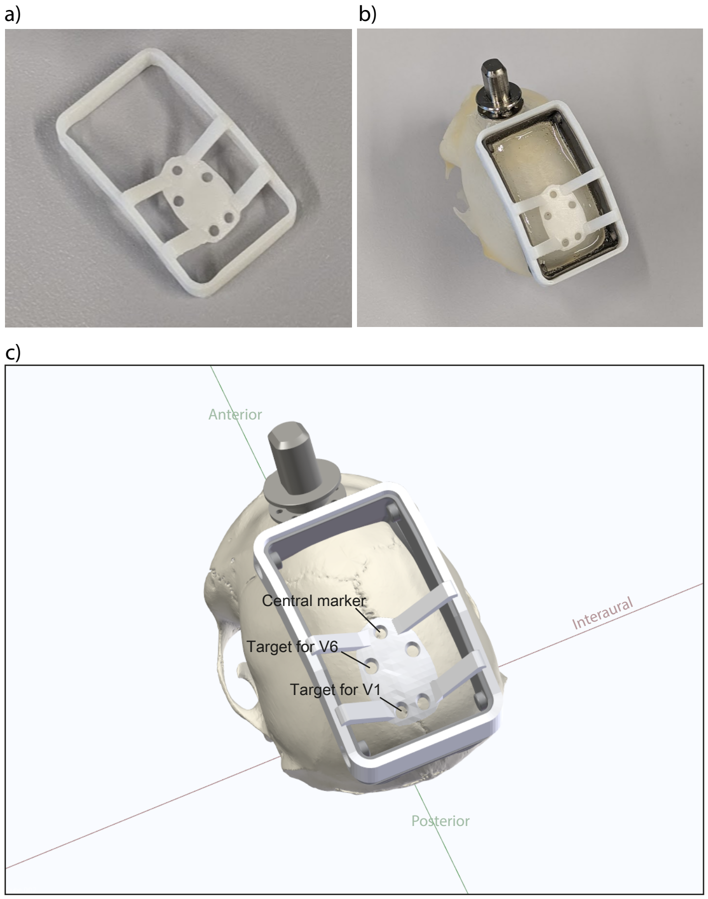


**Supplementary Figure 3 | 3D printed implantation target guide.** Photograph of the implantation guide **(a)** before and **(b)** after placement on the chamber. **c)** 3D rendering of the implantation guide placed on the chamber. The guide hole for the central marker indicates the anterior-posterior and medio-lateral center of the stereotaxic coordinate system. Guide holes for areas V1 and V6 are indicated for the left hemisphere.

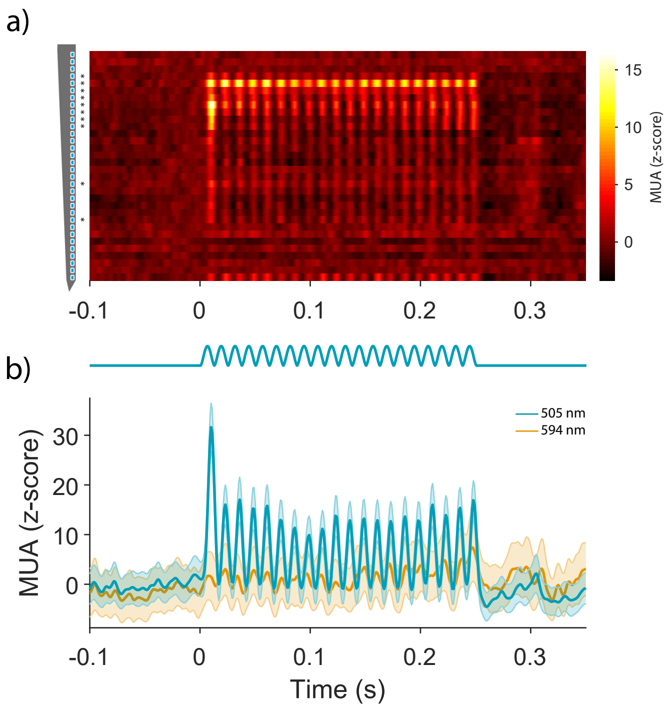


**Supplementary Figure 4 | Optogenetic stimulation with 80 Hz sine wave patterns in Monkey D. a)** Trial averaged, z-scored MUA of all recordings sites from an example shank for the 505 nm stimulation condition. Asterisks on the left indicate significant modulation ($p<0.05$, two-sided Kolmogorov Smirnov test for channels with MUA>3σ) between pre-stimulation baseline window (-25 to 0 ms) and the stimulus window (0 to 25 ms) after laser onset. **b)** Average MUA ±SEM across all significantly modulated channels (n = 10 out of 32 channels), for stimulation with 505 nm and 594 nm, respectively as indicated by the color legend. Stimulation was performed with a peak power of 25 mW for 250 ms. The stimulation waveform is shown between panels. Source data are provided as a Source Data file.

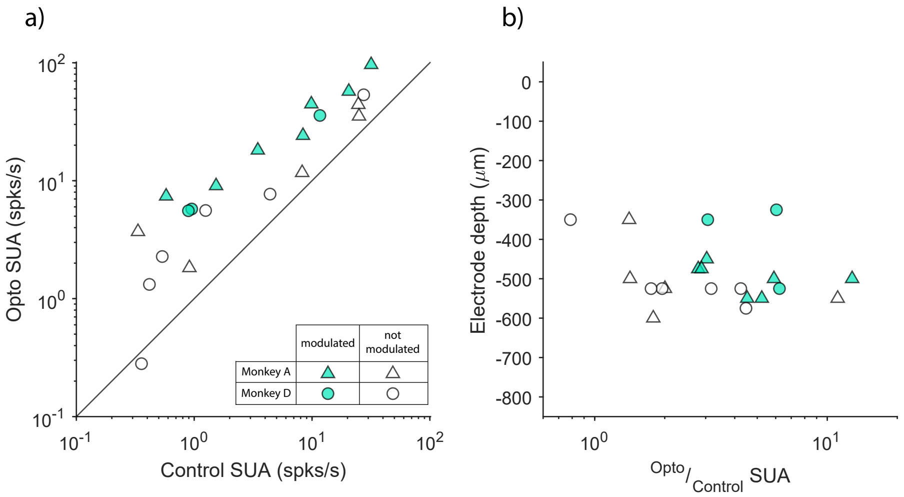


**Supplementary Figure 5 | Single unit population analysis from one optogenetic stimulation session in each animal. a)** Scatter plot of single unit activity (SUA) during stimulation with 505 nm laser vs. control condition (n = 21 units). Filled symbols indicate significant difference between optogenetic stimulation and control condition ($p<0.05$, two-sided Wilcoxon rank sum test for neurons with SUA>3σ over baseline). Across the two animals, 10 out of 21 units (≈ 48%) showed significant modulation. For the control condition in Monkey A, data from trials with 594 nm stimulation and without laser stimulation were pooled (n = 180 control trials; n = 92 opto trials). For Monkey D, the control condition included only data without laser stimulation (n = 84 control trials; n = 178 opto trials). **b)** Magnitude of optogenetic modulation as a function of electrode depth. Most neurons were recorded around the center of the electrode array. No obvious depth pattern with regard to optogenetically induced response strength was observed. Source data are provided as a Source Data file.

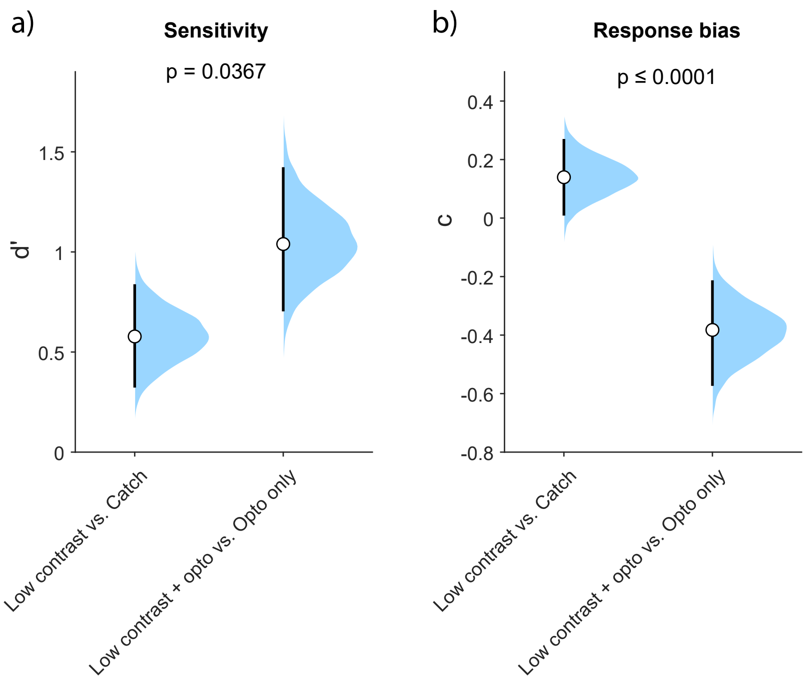


**Supplementary Figure 6 | Behavioral analysis based on signal detection theory. a)** Sensitivity (d') and **b)** response bias (c) calculated from 'low contrast visual' (n = 118) vs. 'catch' trials (n = 467) and from 'low contrast + opto' (n = 109) vs. 'opto only' trials (n = 119). Error bars indicate 95% bootstrap confidence intervals. Blue area shows the probability density estimate of the bootstrapped distribution, truncated at the $0.1^{th}$ and $99.9^{th}$ percentiles. p-values were calculated via a two-sided bootstrap test with 10,000 replications. The smallest possible p-value is therefore 0.0001. Source data are provided as a Source Data file.

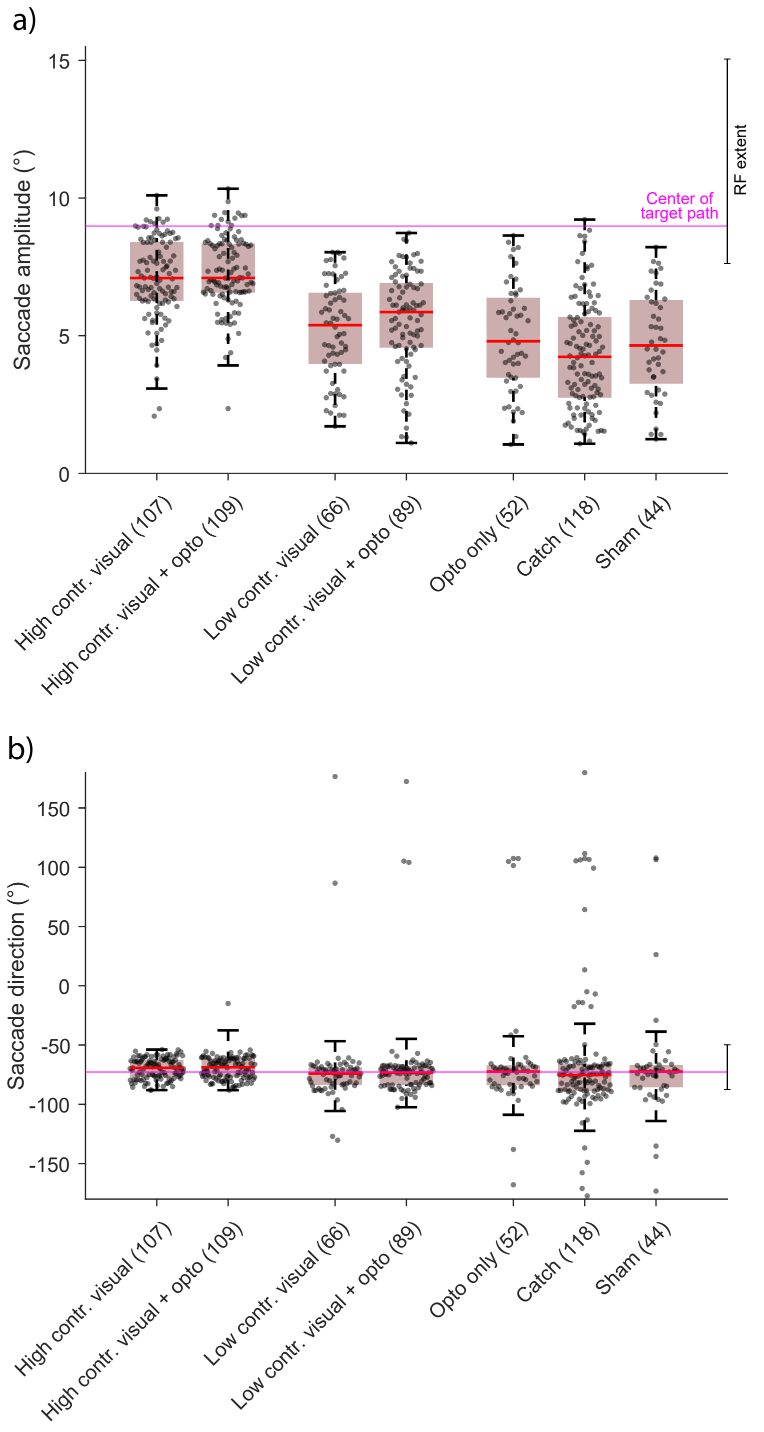


**Supplementary Figure 7 | Saccade analysis of the visual and optogenetic detection task. a)** Saccade amplitude for all behavioral conditions. Numbers of saccades per condition are shown in parenthesis. Gray points indicate values from individual saccades. Box plots show medians (red line), 25th and 75th percentile ranges (red filled box) and 1.5 times the interquartile ranges (whiskers). The magenta colored horizontal line indicates the center of the visual target path. Vertical black line on the right shows mean RF extent (data from Jendritza et al. 2021). The animal showed larger

saccade amplitude for high-contrast visual targets compared to low contrast targets (two-sided Wilcoxon rank sum test; p=6.66e-10). Pairing high-contrast or low-contrast visual targets with optogenetic stimulation did not result in a difference in saccade amplitude (two-sided Wilcoxon rank sum test; p=0.427 and p=0.219, respectively). Saccade amplitude from trials with optogenetic stimulation alone and from sham trials did not differ from catch trials (two-sided Wilcoxon rank sum test; p=0.119 and p=0.219, respectively). **b)** Similar to a) but for saccade direction. Saccade direction from trials with high-contrast vs. low contrast visual targets did not significantly differ (two-sided Watson's $U^2$ test; p=0.0380). Pairing high-contrast or low-contrast visual targets with optogenetic stimulation did not result in a difference in saccade direction (two-sided Watson's $U^2$ test; p= 0.953 and p=0.953, respectively). Saccade direction from trials with optogenetic stimulation alone and from sham trials did not differ from catch trials (Watson's $U^2$ test; p= 0.160 and p=0.355, respectively). Source data are provided as a Source Data file.

**Supplementary Table 1: CT segmentation thresholds.**

| | Thresholds for chamber/cement | Thresholds for screws | Thresholds for skull |
|---|---|---|---|
| **Monkey D:** | 3206.90 - 30111.00 | 11326.50 - 30111.00 | 873.53 - 30111.00 |
| **Monkey U:** | 3206.90 - max | 11326.50 - max | 873.53 - max |
| **Monkey A:** | 3206.90 - max | 11326.50 - max | 1135.69 - max |
