# Supplementary material for: Multi-area recordings and optogenetics in the awake, behaving marmoset
Source: Nat Commun. 2023 Feb 2;14:577. doi: 10.1038/s41467-023-36217-5 (PMC9895452; doi:10.1038/s41467-023-36217-5)
Supplement: Supplementary file 3 — Reporting Summary [file 41467_2023_36217_MOESM3_ESM.pdf]

nature portfolio

Corresponding author(s): Patrick Jendritza
Last updated by author(s): Dec 19, 2022

Reporting Summary

Nature Portfolio wishes to improve the reproducibility of the work that we publish. This form provides structure for consistency and transparency in reporting. For further information on Nature Portfolio policies, see our Editorial Policies and the Editorial Policy Checklist.

Statistics

For all statistical analyses, confirm that the following items are present in the figure legend, table legend, main text, or Methods section.

n/a	Confirmed	
☐	☒	The exact sample size (*n*) for each experimental group/condition, given as a discrete number and unit of measurement
☐	☒	A statement on whether measurements were taken from distinct samples or whether the same sample was measured repeatedly
☐	☒	The statistical test(s) used AND whether they are one- or two-sided<br>*Only common tests should be described solely by name; describe more complex techniques in the Methods section.*
☒	☐	A description of all covariates tested
☐	☒	A description of any assumptions or corrections, such as tests of normality and adjustment for multiple comparisons
☐	☒	A full description of the statistical parameters including central tendency (e.g. means) or other basic estimates (e.g. regression coefficient) AND variation (e.g. standard deviation) or associated estimates of uncertainty (e.g. confidence intervals)
☐	☒	For null hypothesis testing, the test statistic (e.g. *F*, *t*, *r*) with confidence intervals, effect sizes, degrees of freedom and *P* value noted<br>*Give P values as exact values whenever suitable.*
☒	☐	For Bayesian analysis, information on the choice of priors and Markov chain Monte Carlo settings
☒	☐	For hierarchical and complex designs, identification of the appropriate level for tests and full reporting of outcomes
☒	☐	Estimates of effect sizes (e.g. Cohen's *d*, Pearson's *r*), indicating how they were calculated

Our web collection on statistics for biologists contains articles on many of the points above.

Software and code

Policy information about availability of computer code

Data collection	OpenEx suite 2.2 (Tucker Davis Technologies) was used for recording of neural and behavioral data and for generating laser waveforms during optogenetic stimulation. The ARCADE toolbox (https://github.com/esi-neuroscience/ARCADE) for Matlab (Mathworks, version 2014a) was used for real-time behavioral monitoring and control and for stimulus presentation. 3D designs were developed in Blender 2.79 (https://www.blender.org/), OnShape 1.79 (https://www.onshape.com/) and Solidworks 2018 (https://www.solidworks.com/).
Data analysis	Neurophysiological and behavioral data were analyzed in Matlab (Mathworks, version 2020b) and in the 'R' software environment (version 4.0.4). CT data were analyzed in 3D Slicer (https://www.slicer.org, version 4.10.0) and Blender (https://www.blender.org, version 2.79). Custom analysis code is available at https://github.com/PJendritza/MultiAreaOptoMarmo/.

For manuscripts utilizing custom algorithms or software that are central to the research but not yet described in published literature, software must be made available to editors and reviewers. We strongly encourage code deposition in a community repository (e.g. GitHub). See the Nature Portfolio guidelines for submitting code & software for further information.

Data

Policy information about availability of data

All manuscripts must include a data availability statement. This statement should provide the following information, where applicable:

- Accession codes, unique identifiers, or web links for publicly available datasets
- A description of any restrictions on data availability
- For clinical datasets or third party data, please ensure that the statement adheres to our policy

The neural and behavioral data generated in this study have been deposited in the Zenodo repository under accession code https://doi.org/10.5281/zenodo.7259686. Design files for 3D printing and visualization have been deposited in the Zenodo repository under accession code https://doi.org/10.5281/zenodo.7259721. The CT data used to create the template marmoset skull in this study are available in the MorphoSource database under accession code https://doi.org/10.17602/M2/M5203/.

Human research participants

Policy information about studies involving human research participants and Sex and Gender in Research.

Reporting on sex and gender	N/A
Population characteristics	N/A
Recruitment	N/A
Ethics oversight	N/A

Note that full information on the approval of the study protocol must also be provided in the manuscript.

Field-specific reporting

Please select the one below that is the best fit for your research. If you are not sure, read the appropriate sections before making your selection.

☒ Life sciences ☐ Behavioural & social sciences ☐ Ecological, evolutionary & environmental sciences

For a reference copy of the document with all sections, see nature.com/documents/nr-reporting-summary-flat.pdf

Life sciences study design

All studies must disclose on these points even when the disclosure is negative.

Sample size	Sample size was not predetermined. The number of animals was chosen to be consistent with published work that demonstrated the functionality and application of novel methods in awake marmosets (e.g. MacDougall et al. 2016; Johnston et al. 2018; Kondo et al. 2018).
Data exclusions	Noisy epochs/trials in which the standard deviation of multi-unit activity across time was more than 10-times larger than the median standard deviation across all epochs/trials were excluded.
Replication	The novel implantation approach of chamber and headpost was replicated in five animals. Three of these animals were subsequently injected with a viral vector and implanted with electrodes in two areas (6 implantation sites in total). Neural recordings could be successfully replicated in 5 out of the 6 implantation sites. Optogenetic manipulation of behavior was performed in one animal and replicated across multiple sessions.
Randomization	Trial sequences were randomized such that the animals could not predict future trial conditions. No randomization at the level of the animals was performed because the study design did not require multiple groups.
Blinding	No blinding was performed because the study design did not require allocation of multiple groups. Trial sequences were randomized such that neither the experimenters, nor the animals could predict future trial conditions.

Reporting for specific materials, systems and methods

We require information from authors about some types of materials, experimental systems and methods used in many studies. Here, indicate whether each material, system or method listed is relevant to your study. If you are not sure if a list item applies to your research, read the appropriate section before selecting a response.

Materials & experimental systems

n/a	Involved in the study
☒	☐ Antibodies
☒	☐ Eukaryotic cell lines
☒	☐ Palaeontology and archaeology
☐	☒ Animals and other organisms
☒	☐ Clinical data
☒	☐ Dual use research of concern

Methods

n/a	Involved in the study
☒	☐ ChIP-seq
☒	☐ Flow cytometry
☒	☐ MRI-based neuroimaging

Animals and other research organisms

Policy information about studies involving animals; ARRIVE guidelines recommended for reporting animal research, and Sex and Gender in Research

Laboratory animals	Five male common marmosets (Callithrix jacchus) were used in the study. The animals were 33, 47, 57, 59 and 48 months old at the time of first implantation.
Wild animals	The study did not involve wild animals.
Reporting on sex	All animals used in this study were male. The decision to use male animals was due to availability and was not part of the experimental design.
Field-collected samples	The study did not involve samples collected from the field.
Ethics oversight	All animal experiments were approved by the responsible government office (Regierungspräsidium Darmstadt) in accordance with the German law for the protection of animals and the "European Union's Directive 2010/63/EU".

Note that full information on the approval of the study protocol must also be provided in the manuscript.
